# Global Health Security in an Era of Global Health Threats

**DOI:** 10.3201/eid1710.101656

**Published:** 2011-10

**Authors:** Sigfrido Burgos Cáceres

**Affiliations:** Food and Agriculture Organization of the United Nations, Rome, Italy

**Keywords:** policy, health, world health, security measures, security, international health problems, international cooperation, letter

**To the Editor:** Global health security is the protection of the health of persons and societies worldwide. It includes access to medicines, vaccines, and health care, as well as reductions in collective vulnerabilities to global public health events that have the potential to spread across borders. For example, transboundary zoonotic diseases such as avian influenza (H5N1) infections affect animals and humans, thereby threatening health security worldwide because of their high death rates (≈60% in humans) (*1*).

During the past 15 years, fairly standardized responses to threats have been implemented around the globe. Some of these responses have been against severe acute respiratory syndrome and avian influenza (H5N1), which have been overseen by a well-resourced international health system (*2*).

These global health threats have raised the highest levels of political and social concern. This concern has provoked governments and international agencies to address health threats through a security rationale, which emphasizes the themes of national security, biosecurity, and human security. This amalgamation of health issues and security concerns has produced a notion of health security, which is dominated by technical medical approaches and pharmaceutical interventions. These approaches and interventions have already begun to shape the way international health policy is formulated (*3*).

A global vision of health security is very much part of contemporary rhetoric. However, this vision lacks the drive and speed needed to make proposals materialize and operationalize ideas in the geographic areas where they are most desperately needed. Small benefits accrue to members of vulnerable populations who in fact are those most likely to be affected by epidemic diseases. A public health security design that impinges on a global approach runs the risk of neglecting cultural, economic, ecologic, and social conditions on the ground. Regional approaches that address hazards and threats may be more inclusive of context-specific conditions (*4*).

Global public health threats related to infectious pathogens of animal origin are expected to rise. To address these threats, several experts and strategists suggest the initiation of a worldwide early-alerting and -reporting mechanism. Aggregation of disease threats through an event-focused Web-based platform could enable this mechanism. This timely gathering of disease intelligence can inform policymakers about the nature of risks. Disease maps can display details needed to design tailored policies and control measures to tackle diseases according to their specifics (*5*).

Leading scientists and researchers continue to try to understand the global temporal and spatial patterns of animal diseases. This understanding is gained through an array of instruments, ranging from the use of satellite images to cutting-edge molecular technologies. The momentum so far has created an open forum for decisionmakers to collaborate with the leading international agencies to advocate for surveillance, identification, and control of zoonotic diseases to uphold global public health security (*6*).

However, global initiatives suffer from the free-rider problem and from moral hazards. Some low-income countries with weak governance have alerted the international community about their fragile health care systems to capture a nontrivial portion of funds that seldom reach their intended destinations. These resource allocations to developing countries foster aid dependence (*7*).

The international technical agencies tasked with upholding animal and human health should remain at the forefront of identifying and addressing evolving threats. This process will demand continuous flexibility, agility, and a coordinated international effort. Attaining goals of mitigating threats and reducing risks posed by the emergence of zoonoses requires close collaborations with national health authorities and local governments. The large investments planned to improve foresight and prevention might or might not work. If they do not work, apportioning blame to countries or regions for disease flare-ups can result in social, political, cultural, and economic consequences that in the past have turned out to be unjustified, unfair, and ultimately detrimental (*8*).

Clearly, global health threats can be reduced only by the concerted actions of national and international actors. In the years ahead, the international community will almost certainly be expected to bring its formidable technical knowledge, skills, and analytic capabilities to confront this expanded global health threat environment (*9*).

It would be wrong, however, to forget the many insights that current advances in epidemiology and surveillance have delivered. In fact, should the impetus to finance a global health agenda encounter opposition or obstacles, it would seem easier and logical to strengthen already functional activities.

Lastly, the realities and the prevalent policymaking environment have created a trap between a desire to prioritize global health by portraying aspects of it as an existential security issue and the fact that security ultimately might not be the most useful language for describing and institutionalizing the health threats and hazards confronted by societies around the world (*10*). Regardless of whether a trap has been created, action is urgently needed.
